# Supplementary material for: Hot spot prediction in protein-protein interactions by an ensemble system
Source: BMC Syst Biol. 2018 Dec 31;12(Suppl 9):132. doi: 10.1186/s12918-018-0665-8 (PMC6311905; doi:10.1186/s12918-018-0665-8)
Supplement: Supplementary file 2 — All relevant MATLAB code. A simple Matlab implement of our predictor. (ZIP 19,742 kb) [file 12918_2018_665_MOESM2_ESM.zip › Additional file 2/Read ASA/ASA website/dbMPIKT/5C6TL.html]

 NetSurfP - Protein Surface Accessibility and Secondary Structure Predictions

  

|  |  |  |
| --- | --- | --- |
|  |  | NetSurfP - Protein Surface Accessibility and Secondary Structure Predictions  Technical University of Denmark |

  


---

```
# For publication of results, please cite:
# A generic method for assignment of reliability scores applied to solvent accessibility predictions.
# Bent Petersen, Thomas Nordahl Petersen, Pernille Andersen, Morten Nielsen and Claus Lundegaard
# BMC Structural Biology 2009, 9:51 doi:10.1186/1472-6807-9-51
#
# Column 1: Class assignment - B for buried or E for Exposed - Threshold: 25% exposure, but not based on RSA
# Column 2: Amino acid
# Column 3: Sequence name
# Column 4: Amino acid number
# Column 5: Relative Surface Accessibility - RSA
# Column 6: Absolute Surface Accessibility
# Column 7: Z-fit score for RSA prediction
# Column 8: Probability for Alpha-Helix
# Column 9: Probability for Beta-strand
# Column 10: Probability for Coil
E Q  5C6TL                  1    0.752 134.379   0.350   0.003   0.003   0.994
E S  5C6TL                  2    0.379  44.466  -0.197   0.004   0.616   0.381
E V  5C6TL                  3    0.473  72.685   0.966   0.002   0.816   0.182
B L  5C6TL                  4    0.224  40.959  -0.169   0.002   0.816   0.182
E T  5C6TL                  5    0.492  68.171   0.165   0.003   0.718   0.279
B Q  5C6TL                  6    0.190  33.988   0.347   0.004   0.420   0.576
E P  5C6TL                  7    0.421  59.740  -0.253   0.004   0.085   0.910
E P  5C6TL                  8    0.525  74.455  -1.485   0.018   0.047   0.935
E S  5C6TL                  9    0.475  55.635  -2.027   0.019   0.141   0.840
E A  5C6TL                 10    0.353  38.857  -1.004   0.022   0.552   0.426
E S  5C6TL                 11    0.484  56.666  -1.237   0.023   0.655   0.322
E G  5C6TL                 12    0.298  23.445   0.369   0.023   0.655   0.322
E T  5C6TL                 13    0.487  67.491   0.715   0.004   0.420   0.576
E P  5C6TL                 14    0.475  67.388   0.081   0.004   0.085   0.910
E G  5C6TL                 15    0.655  51.572  -1.018   0.005   0.045   0.951
E Q  5C6TL                 16    0.467  83.460   0.386   0.005   0.262   0.733
E R  5C6TL                 17    0.489 111.889   0.927   0.002   0.816   0.182
B V  5C6TL                 18    0.072  11.051   0.311   0.001   0.959   0.040
E T  5C6TL                 19    0.439  60.889   1.536   0.001   0.959   0.040
B I  5C6TL                 20    0.056  10.304   0.370   0.001   0.959   0.040
E S  5C6TL                 21    0.372  43.598   0.964   0.001   0.900   0.099
B C  5C6TL                 22    0.027   3.847   0.214   0.001   0.900   0.099
E S  5C6TL                 23    0.422  49.423   1.245   0.001   0.900   0.099
B G  5C6TL                 24    0.207  16.322  -0.516   0.002   0.816   0.182
B S  5C6TL                 25    0.323  37.879  -0.986   0.004   0.514   0.481
E S  5C6TL                 26    0.510  59.725  -2.030   0.020   0.205   0.775
E S  5C6TL                 27    0.426  49.904  -2.113   0.005   0.262   0.733
E N  5C6TL                 28    0.355  52.001  -1.074   0.005   0.336   0.660
B I  5C6TL                 29    0.235  43.530  -1.152   0.005   0.336   0.660
E E  5C6TL                 30    0.489  85.341  -1.542   0.004   0.197   0.799
E T  5C6TL                 31    0.385  53.386  -1.453   0.004   0.138   0.858
B N  5C6TL                 32    0.192  28.036  -0.683   0.004   0.197   0.799
B Y  5C6TL                 33    0.148  31.713   0.459   0.004   0.616   0.381
B V  5C6TL                 34    0.025   3.796   0.571   0.018   0.846   0.136
B S  5C6TL                 35    0.069   8.122   0.411   0.011   0.918   0.071
B W  5C6TL                 36    0.028   6.662   0.609   0.011   0.918   0.071
B Y  5C6TL                 37    0.053  11.347   1.087   0.011   0.918   0.071
B Q  5C6TL                 38    0.136  24.343   0.972   0.011   0.918   0.071
B Q  5C6TL                 39    0.125  22.379   0.819   0.004   0.616   0.381
E F  5C6TL                 40    0.392  78.594   1.679   0.004   0.085   0.910
E P  5C6TL                 41    0.559  79.336   0.087   0.005   0.015   0.979
E G  5C6TL                 42    0.830  65.321  -2.119   0.003   0.003   0.994
E T  5C6TL                 43    0.418  58.032   0.743   0.018   0.047   0.935
E A  5C6TL                 44    0.346  38.129   0.094   0.019   0.141   0.840
B P  5C6TL                 45    0.096  13.594   0.735   0.004   0.420   0.576
B K  5C6TL                 46    0.263  54.099   0.496   0.002   0.816   0.182
B L  5C6TL                 47    0.120  21.935   0.363   0.001   0.959   0.040
B L  5C6TL                 48    0.028   5.200   0.655   0.001   0.959   0.040
B I  5C6TL                 49    0.035   6.420  -0.557   0.000   0.983   0.017
B Y  5C6TL                 50    0.121  25.879  -0.603   0.001   0.959   0.040
B R  5C6TL                 51    0.129  29.633  -0.151   0.001   0.900   0.099
B N  5C6TL                 52    0.152  22.209  -0.860   0.004   0.616   0.381
B N  5C6TL                 53    0.286  41.812  -0.780   0.005   0.262   0.733
E Q  5C6TL                 54    0.550  98.159  -1.154   0.004   0.197   0.799
E R  5C6TL                 55    0.423  96.844  -0.329   0.018   0.088   0.893
E P  5C6TL                 56    0.558  79.194  -1.723   0.018   0.047   0.935
E S  5C6TL                 57    0.733  85.872  -1.153   0.018   0.047   0.935
E G  5C6TL                 58    0.596  46.905  -1.108   0.018   0.088   0.893
B V  5C6TL                 59    0.201  30.909  -0.053   0.004   0.138   0.858
E P  5C6TL                 60    0.578  81.976  -0.149   0.004   0.138   0.858
E D  5C6TL                 61    0.578  83.347  -2.110   0.005   0.262   0.733
E R  5C6TL                 62    0.344  78.890  -0.244   0.004   0.616   0.381
B F  5C6TL                 63    0.149  29.864  -0.712   0.002   0.816   0.182
E S  5C6TL                 64    0.282  33.097  -0.856   0.002   0.816   0.182
B G  5C6TL                 65    0.310  24.428  -2.093   0.002   0.816   0.182
E S  5C6TL                 66    0.387  45.321  -1.067   0.004   0.616   0.381
E K  5C6TL                 67    0.451  92.791  -0.821   0.004   0.420   0.576
E S  5C6TL                 68    0.577  67.648  -1.460   0.004   0.138   0.858
E G  5C6TL                 69    0.613  48.259  -1.249   0.005   0.045   0.951
E T  5C6TL                 70    0.367  50.917  -0.939   0.005   0.262   0.733
E S  5C6TL                 71    0.338  39.625   0.063   0.002   0.816   0.182
B A  5C6TL                 72    0.085   9.389   0.171   0.001   0.959   0.040
B S  5C6TL                 73    0.206  24.178   0.592   0.001   0.959   0.040
B L  5C6TL                 74    0.034   6.152   0.296   0.001   0.959   0.040
B A  5C6TL                 75    0.194  21.401  -0.019   0.001   0.959   0.040
B I  5C6TL                 76    0.050   9.195   0.193   0.001   0.959   0.040
E S  5C6TL                 77    0.329  38.535   0.295   0.002   0.816   0.182
E G  5C6TL                 78    0.380  29.914  -0.994   0.004   0.514   0.481
B L  5C6TL                 79    0.183  33.452  -0.112   0.005   0.336   0.660
E R  5C6TL                 80    0.485 110.950   0.887   0.005   0.336   0.660
E S  5C6TL                 81    0.402  47.126  -0.405   0.022   0.359   0.619
E E  5C6TL                 82    0.644 112.454  -0.782   0.020   0.205   0.775
E D  5C6TL                 83    0.493  71.070  -0.556   0.019   0.141   0.840
E E  5C6TL                 84    0.346  60.499  -1.055   0.022   0.359   0.619
B A  5C6TL                 85    0.248  27.341  -1.893   0.004   0.514   0.481
E E  5C6TL                 86    0.344  60.079   0.005   0.003   0.718   0.279
B Y  5C6TL                 87    0.059  12.566   0.825   0.003   0.718   0.279
B Y  5C6TL                 88    0.159  34.085   0.764   0.002   0.816   0.182
B C  5C6TL                 89    0.096  13.408  -0.977   0.002   0.816   0.182
B G  5C6TL                 90    0.221  17.369  -0.139   0.003   0.718   0.279
B T  5C6TL                 91    0.168  23.343  -0.534   0.022   0.359   0.619
B W  5C6TL                 92    0.255  61.279  -1.188   0.022   0.359   0.619
E D  5C6TL                 93    0.380  54.816  -1.782   0.021   0.279   0.699
E D  5C6TL                 94    0.464  66.819  -1.914   0.018   0.088   0.893
B N  5C6TL                 95    0.300  43.847  -1.852   0.019   0.141   0.840
E S  5C6TL                 96    0.342  40.047  -1.334   0.021   0.279   0.699
E W  5C6TL                 97    0.341  81.962  -1.214   0.022   0.359   0.619
B V  5C6TL                 98    0.303  46.556  -1.543   0.021   0.279   0.699
B F  5C6TL                 99    0.194  39.036  -1.640   0.020   0.205   0.775
B G  5C6TL                100    0.309  24.303  -2.633   0.019   0.141   0.840
E G  5C6TL                101    0.375  29.536  -1.913   0.018   0.088   0.893
E G  5C6TL                102    0.347  27.317  -1.969   0.019   0.141   0.840
E T  5C6TL                103    0.321  44.523  -1.165   0.021   0.279   0.699
E K  5C6TL                104    0.300  61.689  -1.243   0.021   0.451   0.528
B L  5C6TL                105    0.281  51.433  -0.703   0.021   0.451   0.528
E T  5C6TL                106    0.319  44.218  -0.616   0.021   0.451   0.528
E V  5C6TL                107    0.387  59.436  -1.080   0.022   0.359   0.619
E L  5C6TL                108    0.331  60.643  -0.871   0.021   0.279   0.699
E G  5C6TL                109    0.477  37.564  -1.256   0.021   0.279   0.699
E Q  5C6TL                110    0.372  66.457  -0.950   0.021   0.279   0.699
E P  5C6TL                111    0.476  67.488  -1.263   0.056   0.142   0.802
E K  5C6TL                112    0.358  73.641  -0.723   0.056   0.142   0.802
E A  5C6TL                113    0.422  46.482  -0.996   0.118   0.150   0.732
E A  5C6TL                114    0.397  43.793  -1.077   0.056   0.142   0.802
E P  5C6TL                115    0.389  55.227  -1.695   0.056   0.142   0.802
E S  5C6TL                116    0.362  42.450  -1.408   0.064   0.216   0.721
E V  5C6TL                117    0.359  55.178  -1.429   0.056   0.142   0.802
E T  5C6TL                118    0.392  54.329  -1.606   0.052   0.084   0.864
E L  5C6TL                119    0.416  76.096  -2.447   0.018   0.088   0.893
B F  5C6TL                120    0.310  62.237  -1.704   0.018   0.047   0.935
E P  5C6TL                121    0.346  49.126  -1.700   0.005   0.015   0.979
B P  5C6TL                122    0.322  45.763  -2.704   0.005   0.015   0.979
B S  5C6TL                123    0.239  27.964  -2.611   0.005   0.045   0.951
E S  5C6TL                124    0.356  41.700  -1.782   0.018   0.088   0.893
E E  5C6TL                125    0.551  96.172  -1.960   0.018   0.088   0.893
E E  5C6TL                126    0.405  70.719  -2.091   0.018   0.088   0.893
B L  5C6TL                127    0.241  44.200  -1.265   0.005   0.015   0.979
E Q  5C6TL                128    0.534  95.301  -1.151   0.018   0.019   0.964
E A  5C6TL                129    0.665  73.305  -1.078   0.003   0.003   0.994
E N  5C6TL                130    0.547  80.095  -1.598   0.005   0.015   0.979
E K  5C6TL                131    0.419  86.188   0.121   0.005   0.262   0.733
B A  5C6TL                132    0.066   7.251  -0.254   0.002   0.816   0.182
B T  5C6TL                133    0.188  26.131   1.015   0.001   0.959   0.040
B L  5C6TL                134    0.039   7.232   0.271   0.001   0.959   0.040
B V  5C6TL                135    0.040   6.163   0.344   0.001   0.959   0.040
B C  5C6TL                136    0.020   2.864   1.045   0.001   0.959   0.040
B L  5C6TL                137    0.132  24.169   0.694   0.001   0.959   0.040
B I  5C6TL                138    0.037   6.919  -0.444   0.001   0.900   0.099
B S  5C6TL                139    0.283  33.179   0.158   0.004   0.616   0.381
E D  5C6TL                140    0.530  76.416  -1.456   0.004   0.197   0.799
B F  5C6TL                141    0.258  51.761  -1.413   0.004   0.138   0.858
E Y  5C6TL                142    0.320  68.277  -0.509   0.004   0.197   0.799
B P  5C6TL                143    0.274  38.895  -1.546   0.004   0.197   0.799
E G  5C6TL                144    0.548  43.143  -1.437   0.004   0.197   0.799
E A  5C6TL                145    0.344  37.931  -1.174   0.004   0.616   0.381
B V  5C6TL                146    0.090  13.787   0.384   0.001   0.900   0.099
B T  5C6TL                147    0.235  32.664   0.188   0.001   0.959   0.040
B V  5C6TL                148    0.043   6.624   0.256   0.001   0.959   0.040
B A  5C6TL                149    0.219  24.167   1.032   0.011   0.918   0.071
B W  5C6TL                150    0.048  11.544   0.110   0.011   0.918   0.071
E K  5C6TL                151    0.339  69.712   1.178   0.002   0.816   0.182
E A  5C6TL                152    0.420  46.339   0.983   0.005   0.336   0.660
E D  5C6TL                153    0.769 110.798   0.400   0.005   0.015   0.979
E S  5C6TL                154    0.791  92.740  -0.895   0.005   0.015   0.979
E S  5C6TL                155    0.606  71.082  -0.598   0.004   0.197   0.799
E P  5C6TL                156    0.552  78.286  -0.729   0.005   0.262   0.733
E V  5C6TL                157    0.357  54.825  -0.021   0.020   0.205   0.775
E K  5C6TL                158    0.561 115.377  -0.845   0.020   0.205   0.775
E A  5C6TL                159    0.537  59.210  -1.748   0.018   0.088   0.893
E G  5C6TL                160    0.622  48.951  -2.318   0.018   0.088   0.893
E V  5C6TL                161    0.346  53.211  -0.964   0.021   0.279   0.699
E E  5C6TL                162    0.467  81.567  -0.880   0.022   0.552   0.426
E T  5C6TL                163    0.474  65.730  -1.396   0.021   0.451   0.528
E T  5C6TL                164    0.412  57.089  -1.213   0.021   0.279   0.699
B T  5C6TL                165    0.253  35.133  -0.117   0.020   0.205   0.775
E P  5C6TL                166    0.309  43.918  -0.888   0.021   0.279   0.699
E S  5C6TL                167    0.364  42.719  -1.877   0.022   0.552   0.426
E K  5C6TL                168    0.390  80.285  -0.444   0.023   0.655   0.322
E Q  5C6TL                169    0.287  51.187  -1.162   0.004   0.514   0.481
E S  5C6TL                170    0.601  70.390  -1.403   0.004   0.138   0.858
E N  5C6TL                171    0.782 114.412  -2.674   0.005   0.015   0.979
E N  5C6TL                172    0.475  69.569  -2.100   0.005   0.045   0.951
E K  5C6TL                173    0.359  73.908  -0.396   0.004   0.514   0.481
B Y  5C6TL                174    0.173  36.885  -1.432   0.003   0.718   0.279
B A  5C6TL                175    0.166  18.249  -1.187   0.002   0.816   0.182
B A  5C6TL                176    0.171  18.888  -0.678   0.001   0.900   0.099
B S  5C6TL                177    0.204  23.874  -0.702   0.001   0.900   0.099
B S  5C6TL                178    0.058   6.821  -0.692   0.001   0.959   0.040
B Y  5C6TL                179    0.183  39.086   0.042   0.001   0.959   0.040
B L  5C6TL                180    0.048   8.844  -0.018   0.001   0.959   0.040
E S  5C6TL                181    0.282  33.004   0.775   0.001   0.900   0.099
B L  5C6TL                182    0.053   9.631   0.698   0.002   0.816   0.182
E T  5C6TL                183    0.333  46.173   1.285   0.004   0.420   0.576
E P  5C6TL                184    0.420  59.584  -0.702   0.021   0.279   0.699
E E  5C6TL                185    0.558  97.448  -0.776   0.020   0.205   0.775
E Q  5C6TL                186    0.529  94.497   0.721   0.021   0.279   0.699
B W  5C6TL                187    0.190  45.743  -0.892   0.020   0.205   0.775
E K  5C6TL                188    0.685 140.925  -0.230   0.004   0.085   0.910
E S  5C6TL                189    0.604  70.789   0.476   0.005   0.045   0.951
E H  5C6TL                190    0.618 112.414  -0.554   0.005   0.045   0.951
E R  5C6TL                191    0.577 132.179   0.508   0.005   0.336   0.660
E S  5C6TL                192    0.454  53.162   1.788   0.002   0.816   0.182
B Y  5C6TL                193    0.067  14.297   0.876   0.001   0.959   0.040
E S  5C6TL                194    0.325  38.125   1.897   0.001   0.959   0.040
B C  5C6TL                195    0.024   3.356   0.846   0.001   0.959   0.040
E Q  5C6TL                196    0.318  56.759   1.407   0.001   0.959   0.040
B V  5C6TL                197    0.076  11.712  -0.191   0.001   0.900   0.099
E T  5C6TL                198    0.477  66.118   0.752   0.001   0.900   0.099
B H  5C6TL                199    0.152  27.612   0.561   0.003   0.718   0.279
E E  5C6TL                200    0.678 118.482   0.375   0.005   0.045   0.951
E G  5C6TL                201    0.757  59.592  -1.265   0.005   0.015   0.979
E S  5C6TL                202    0.590  69.183  -0.157   0.004   0.197   0.799
E T  5C6TL                203    0.557  77.298   0.413   0.004   0.420   0.576
E V  5C6TL                204    0.404  62.110   0.535   0.003   0.718   0.279
E E  5C6TL                205    0.545  95.264   0.240   0.002   0.816   0.182
E K  5C6TL                206    0.381  78.392   1.364   0.001   0.900   0.099
E T  5C6TL                207    0.521  72.263   0.521   0.001   0.900   0.099
B V  5C6TL                208    0.129  19.843   1.339   0.001   0.900   0.099
E A  5C6TL                209    0.533  58.781   1.370   0.003   0.718   0.279
E P  5C6TL                210    0.436  61.812   0.401   0.004   0.197   0.799
E T  5C6TL                211    0.630  87.409  -1.007   0.019   0.141   0.840
E E  5C6TL                212    0.582 101.710   0.674   0.019   0.141   0.840
B C  5C6TL                213    0.201  28.263  -1.551   0.018   0.047   0.935
E S  5C6TL                214    0.762  89.318  -0.531   0.003   0.003   0.994

Explain the output.  Go back.
```

---
